# Creation of a multi-segmented optical needle with prescribed length and spacing using the radiation pattern from a sectional-uniform line source

**DOI:** 10.1038/s41598-017-11501-9

**Published:** 2017-09-06

**Authors:** Yanzhong Yu, Han Huang, Mianmian Zhou, Qiwen Zhan

**Affiliations:** 1 College of Physics & Information Engineering, Quanzhou Normal University, Quanzhou, Fujian, 362000 China; 2 Department of Electro-Optics and Photonics, University of Dayton, 300 College Park, Dayton, Ohio, 45469 USA

## Abstract

This paper presents a method to generate a multi-segmented optical needle with a strong longitudinally polarized field, uniform intensity along the optical axis, and a transverse size (~0.36λ). The length of each segment in the optical needle and the spacing between adjacent segments are controllable by reversing and focusing the radiation pattern from a sectional-uniform line source antenna to the focal volume of a 4Pi focusing system. By solving the inverse problem, we can obtain the required incident field distribution at the pupil plane to create the multi-segmented optical needle. Numerical examples demonstrate that a multi-segmented optical needle with variable focal depth, adjustable interval, narrow lateral width, homogeneous intensity, and high longitudinal polarization purity can be formed using the proposed approach. The length of each needle segment is approximately equal to the length of the corresponding sectional uniform line source. The multi-segmented optical needle may be employed in applications such as multi-particle acceleration, multi-particle trapping and manipulation, laser machining, and laser material processing.

## Introduction

In the past decade, the high-numerical-aperture (NA) focusing of a cylindrical vector beam has attracted considerable interest because of its novel focusing property^1–5^. A number of unique focal field distributions, such as spherical spot^6–8^, optical chain^9, 10^, flattop focus^11^, light cage^12^ and so on. In particular, notable attention has been devoted to the generation of light needles with ultra-long depth of focus (DOF), narrow radial width, uniform intensity, and high-purity polarization state^13, 14^. Many methods have been proposed to create optical needles with these properties. For example, by focusing a radially polarized Bessel–Gaussian beam with a high-NA lens and a diffractive optical element (DOE), a longitudinally polarized needle with 0.43λ beam size and approximately 4λ DOF was first obtained by Wang *et al*.^15^. An ultra-long light needle (~14λ) with a strong transversally polarized field, uniform intensity along the optical axis and a subwavelength beam size (~0.9λ) was obtained by focusing hybrid polarized vector beams through a dielectric interface under an annular high-NA lens^16^. A high-NA Fresnel zone plate (FZP) illuminated by a radially polarized vector beam was used to form a super-Gaussian optical needle with 0.366λ beam size and a strong longitudinally polarized field^17^. By modulating the Bessel-Gaussian radially polarized vector beam using the cosine synthesized filter under a reflection parabolic mirror system, a super-Gaussian optical needle with the minimal spot size (0.36λ) and pure longitudinal polarization was generated^13^. To the best of our knowledge, the realized needle is only a single segment. However, a multi-segmented light needle may be desirable in applications such as multi-particle acceleration^18^, multi-particle trapping and manipulation^19, 20^. In this letter, we report a simple and flexible method to produce a multi-segmented optical needle with tunable DOF and interval between adjacent segments. This goal can be achieved by inversing and focusing the field eradiated from a sectional-uniform line source antenna to the vicinity of the focus in a 4Pi focusing system.

## Results

### Proposed scheme

**Figure 1:**
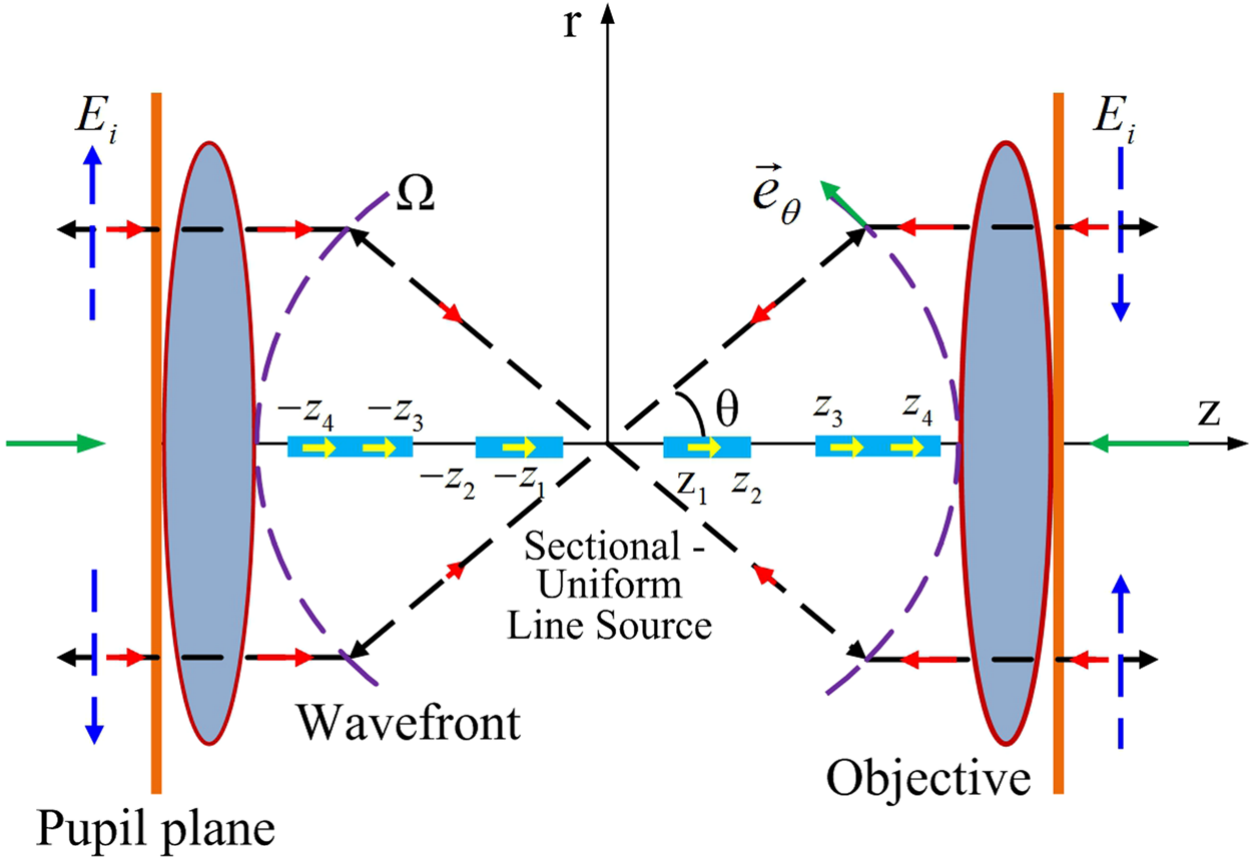
Schematic of a 4Pi system constructed by two confocal high-NA objective lenses. A sectional-uniform line source antenna, along which the current is constant, is centered at the foci of two high-NA objectives and aligned along the optical axis (denoted by yellow arrows). The field radiated from the antenna (denoted by black arrows) is completely gathered by two identical objective lenses to their pupil planes. Next, the field at the pupil planes is inversely propagated (denoted by red arrows) with a relative π phase shift (denoted by blue arrows) and focused by the 4Pi focusing system.

The schematic setup of a 4Pi focusing system to generate the multi-segmented optical needle is shown in Fig. 1. Let us suppose that the sectional-uniform line source antenna (denoted by yellow arrows in Fig. 1) centered at the focus of the 4Pi system, which consists of two high-NA objective lenses, is aligned along the optical axis. Thus, the current is^21^

1 $$I(z\text{'})=\{\begin{array}{ll}{I}_{0} & x\text{'}=0,{\rm{y}}\text{'}=0,\,{z}_{1}\le |z\text{'}|\le {z}_{2},{z}_{3}\le |z\text{'}|\le {z}_{4},{z}_{2n-1}\le |z\text{'}|\le {z}_{2n}\\ 0 & elsewhere\end{array},$$

where ${I}_{0}$ is the electric current constant and ${z}_{2n-1}$ and ${z}_{2n}$ denote the beginning and ending points of the *n* ^*th*^ section uniform line source, respectively, along which the current is constant. The length of the *n* ^*th*^ section is ${L}_{n}={z}_{2n}-{z}_{2n-1}$, and the *n* ^*th*^ blanking between contiguous sections is ${S}_{n}={z}_{2n+1}-{z}_{2n}$. The electromagnetic field radiated from this antenna in the far zone is^21^

2 $$\begin{array}{rcl}\vec{F}(\theta ) & = & C\,\tan \,\theta [\sin ({z}_{2}\beta \,\cos \,\theta )-\,\sin ({z}_{1}\beta \,\cos \,\theta )\\  &  & +\,\sin ({z}_{4}\beta \,\cos \,\theta )-\,\sin ({z}_{3}\beta \,\cos \,\theta )\\  &  & +\,\sin ({z}_{2n}\beta \,\cos \,\theta )-\,\sin ({z}_{2n-1}\beta \,\cos \,\theta )]{\vec{e}}_{\theta }\\  & = & CK(\theta ){\vec{e}}_{\theta }\end{array},$$

where $C$ is the radiation coefficient for the sectional-uniform line source; *β* and *θ* are the wave number and radiation angle between the radiation direction and the z-axis, respectively; and ${\vec{e}}_{\theta }$ is a unit vector along the $\theta$ direction. If the radiation field $\vec{F}(\theta )$ (denoted by black arrows in Fig. 1) is completely collected at the pupil planes by two high-NA objective lenses and subsequently reversely propagated (denoted by red arrows in Fig. 1) with a relative π phase shift to the focus region (denoted by blue arrows in Fig. 1), we obtain the focal field distributions, which are calculated according to Richards-Wolf vectorial diffraction method^22, 23^ as

3 $${E}_{r}(r,\varphi ,z)=C{\int }_{0}^{{\theta }_{\max }}K(\theta )\sin \,\theta \,\cos \,\theta {J}_{1}(kr\,\sin \,\theta )\exp (ikz\,\cos \,\theta )d\theta ,$$

4 $${E}_{z}(r,\varphi ,z)=jC{\int }_{0}^{{\theta }_{\max }}K(\theta ){\sin }^{2}\theta {J}_{0}(kr\,\sin \,\theta )\exp (ikz\,\cos \,\theta )d\theta ,$$

The required illumination at the pupil plane to create such focal field can be analytically obtained from the radiation field $\vec{F}(\theta )$ in Eq. (1) and Richards-Wolf theory^22, 23^. This incident field distribution ${\vec{E}}_{i}(r)$ at the pupil plane of the high-NA objective lens, which obeys the Helmholtz condition of the ray projection function $g(\theta )=\tan \,\theta$, may be computed by^7^

5 $${\vec{E}}_{i}(r)=\vec{F}(\theta ){(\sqrt{\cos \theta })}^{3},$$

where $r=f\,\tan \,\theta$, and $r$ is the radial position in the pupil plane and $f$ is the focal length of the 4Pi focusing system.

### Generation of a multi-segmented optical needle

**Figure 2:**
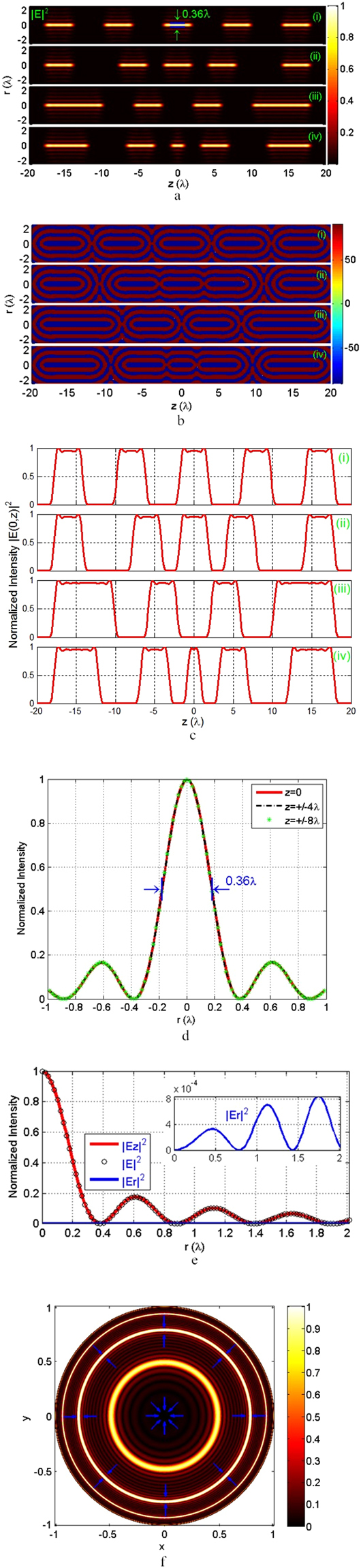
Generation of a multi-segmented optical needle with prescribed length and spacing. (**a**) Total intensities $|E{|}^{2}$ in the r-z plane; (**b**) corresponding phase distributions of the ${E}_{z}$ component. (**c**) Axial intensities $|E(0,z){|}^{2}$ for multi-segmented light needles with (i) identical lengths and intervals: ${{\rm{z}}}_{1}=0$, ${{\rm{z}}}_{2}=2\lambda$, ${{\rm{z}}}_{3}=6\lambda$, ${{\rm{z}}}_{4}=10\lambda$, ${{\rm{z}}}_{5}=14\lambda$, ${{\rm{z}}}_{6}=18\lambda$; (ii) identical lengths and different intervals: ${{\rm{z}}}_{1}=0$, ${{\rm{z}}}_{2}=2\lambda$, ${{\rm{z}}}_{3}=4\lambda$, ${{\rm{z}}}_{4}=8\lambda$, ${{\rm{z}}}_{5}=14\lambda$, ${{\rm{z}}}_{6}=18\lambda$; (iii) different lengths and identical intervals: ${{\rm{z}}}_{1}=2\lambda$, ${{\rm{z}}}_{2}=6\lambda$, ${{\rm{z}}}_{3}=10\lambda$, ${{\rm{z}}}_{4}=18\lambda$; and (iv) different lengths and intervals: ${{\rm{z}}}_{1}=0$, ${{\rm{z}}}_{2}=2\lambda$, ${{\rm{z}}}_{3}=3\lambda$, ${{\rm{z}}}_{4}=7\lambda$, ${{\rm{z}}}_{5}=12\lambda$, ${{\rm{z}}}_{6}=18\lambda$. The transverse intensity along the ${\rm{z}}=0,\pm 4\lambda ,\pm {\rm{8}}\lambda$, polarization structure at the ${\rm{z}}=\pm \lambda$ plane, required input field distribution at the normalized pupil plane to create a multi-segmented light needle, as shown in Fig. 2a(i), are plotted in (**d**), (**e**) and (**f**), respectively.

Since the radiation coefficient $C$ is unrelated to the shape of the focusing field, it is normalized to 1 in our calculation. The numerical aperture is set as $NA=1.0$(corresponding to ${\theta }_{\max }=\pi /2$), which is achievable from the report^24, 25^, to converge the total eradiation field. Using Eqs (3) and (4), one obtains the multi-segmented optical needle with variable length and spacing near the focus volume. In this study, we provide four examples to validate the proposed method. Figure 2 a–c illustrate the total intensities ${|E|}^{2}={|{E}_{r}|}^{2}+|{E}_{z}{|}^{2}$ in the r-z plane, corresponding phase distributions of the ${E}_{z}$ component, and axial intensities $|E(0,z){|}^{2}$ for (i) identical lengths and intervals; (ii) identical lengths and different intervals; (iii) different lengths and identical intervals; and (iv) different lengths and intervals. d_FWHM_ and z_FWHM_ are introduced to characterize the transverse beam size and axial depth of focus, respectively^13, 14^. For all cases, d_FWHM_ = $0.36\lambda$, which is currently the smallest achievable beam size. Additionally, d_FWHM_ is independent of ${L}_{n}$ and ${S}_{n}$ and remains unchanged in the range of each segment light needle, as observed in Fig. 2d. The *n* ^*th*^ z_FWHM_ is approximately equal to length ${L}_{n}$ and only determined by ${L}_{n}$. The *n* ^*th*^ spacing between two adjacent segment needles is approximately equal to the blanking ${S}_{n}$. Figure 2b shows that the phase distribution of the ${E}_{z}$ component exhibits a binary behavior between −90^°^ and +90°; in particular, the value of −90° is maintained in the scope of the *n* ^*th*^ main lobe, whose axial length is equal to the length of ${L}_{n}$. The phase stability must be maintained to generate a high-quality light needle field^26^. The non-uniformity of the axial intensity^27^ in every segment needle is below 3.4%. Thus, the axial intensity is uniform, as shown in Fig. 2c. The evaluated polarization purity^28^ of each needle is more than 98%, which implies that the produced multi-segmented needle is a high-purity longitudinally polarized field, as illustrated in Fig. 2e. The required incident field ${\vec{E}}_{i}(r)$ at the normalized pupil plane to create such optical needle is obtained from Eq. (5). Obviously, ${\vec{E}}_{i}(r)$ is a spatially modulated radial polarization field with annular bright belts separated by dark rings (Fig. 2 f). This input field may be achievable at present using the latest technologies of spatial light modulation and metasurfaces^29–31^.

## Conclusions

We demonstrated that a multi-segmented optical needle with adjustable length and spacing can be easily achieved by reversing and focusing the field emitted from the sectional-uniform line source antenna to the focus of the 4Pi focusing system, which is formed by two confocal high-NA objective lenses. The generated light needle with a sectionally homogeneous intensity along the optical axis is a strong longitudinally polarized field. The *n* ^*th*^ z_FWHM_ and *n* ^*th*^ spacing are approximately equal to length ${L}_{n}$ and blanking ${S}_{n}$, respectively. d_FWHM_ of $0.36\lambda$ does not rely on the parameters of ${L}_{n}$ and ${S}_{n}$. The phenomenon of a binary phase between −90° and +90° can be observed from the dominant longitudinal component ${E}_{z}$. This multi-segmented light needle may be notably suitable for promising applications in multi-particle confinement, manipulation and laser direct writing.
